# Integrated programs for mothers with substance abuse issues: A systematic review of studies reporting on parenting outcomes

**DOI:** 10.1186/1477-7517-9-14

**Published:** 2012-03-19

**Authors:** Alison Niccols, Karen Milligan, Wendy Sword, Lehana Thabane, Joanna Henderson, Ainsley Smith

**Affiliations:** 1 Department of Psychiatry and Behavioural Neurosciences, McMaster University, 280 Holbrook Building, McMaster Children's Hospital-Chedoke Site, Hamilton, Ontario, Box 2000 L9N 3Z5, Canada; 2 Integra, 25 Imperial Street, Toronto, Ontario M5P 1B9, Canada; 3 School of Nursing, McMaster University, 1200 Main Street West, Room 3N25G, Hamilton, Ontario L8N 3Z5, Canada; 4 Department of Clinical Epidemiology and Biostatistics, McMaster University, St. Joseph's Healthcare, 50 Charlton Avenue., 3rd Floor, Room H325, Hamilton, Ontario L8N 4A6, Canada; 5 Centre for Addiction and Mental Health, Department of Psychiatry, University of Toronto, 250 College Street, Toronto, Ontario M5T 1R8, Canada; 6 Department of Psychiatry and Behavioural Neurosciences, McMaster University, 284 Holbrook Building, McMaster Children's Hospital-Chedoke Site, Hamilton, Ontario, Box 2000 L9N 3Z5, Canada

**Keywords:** Women, Mothers, Substance use, Parenting, Integrated programs

## Abstract

**Background:**

Integrated treatment programs (those that include on-site pregnancy-, parenting-, or child-related services with addiction services) were developed to break the intergenerational cycle of addiction, dysfunctional parenting, and poor outcomes for mothers and children, yet there has been no systematic review of studies of parenting outcomes.

**Objectives:**

As part of larger systematic review to examine the effectiveness of integrated programs for mothers with substance abuse issues, we performed a systematic review of studies published from 1990 to 2011 with data on parenting outcomes.

**Methods:**

Literature search strategies included online bibliographic database searches, checking printed sources, and requests to researchers. Studies were included if all participants were mothers with substance abuse problems at baseline, the treatment program included at least one specific substance use treatment and at least one parenting or child service, and there were quantitative data on parenting outcomes. We summarized data on parenting skills and capacity outcomes.

**Results:**

There were 24 cohort studies, 3 quasi-experimental studies, and 4 randomized trials. In the three randomized trials comparing integrated programs to addiction treatment-as-usual (*N *= 419), most improvements in parenting skills favored integrated programs and most effect sizes indicated that this advantage was small, *d*s = -0.02 to 0.94. Results for child protection services involvement did not differ by group. In the three studies that examined factors associated with treatment effects, parenting improvements were associated with attachment-based parenting interventions, children residing in the treatment facility, and improvements in maternal mental health.

**Conclusions:**

This is the first systematic review of studies evaluating the effectiveness of integrated programs on parenting. The limited available evidence supports integrated programs, as findings suggest that they are associated with improvements in parenting skills. However, more research is required comparing integrated programs to addiction treatment-as-usual. This review highlights the need for improved methodology, study quality, and reporting to improve our understanding of how best to meet the parenting needs of women with substance abuse issues.

## Background

Substance abuse among women is a serious problem for parenting and represents considerable human and financial burden to society. Estimates suggest that 50-80% of child welfare cases involve a parent who abuses alcohol or other drugs and mothers make up the majority of substance-abusing parents in the child welfare system [1,2]. In the United States, up to 70% of women in substance abuse treatment have children [2]. Rates of substance abuse in women have been increasing [3] and substance abuse in women also is associated with a unique constellation of risk factors and needs, including greater vulnerability to adverse physiological consequences than men, greater prevalence of mental health problems, histories of physical or sexual abuse, serious medical problems, poor nutrition, relationship problems including domestic violence, and deficits in social support [4,5]. The unique risk factors and presenting needs of women have resulted in the development of women-specific comprehensive treatment models [3]. However, in addition to having gender-specific needs, women with substance abuse issues also have unique needs as mothers.

Research has shown that women who abuse substances may have difficulties providing stable, nurturing environments for their children compounded by challenging life circumstances, including severe economic and social problems, such as lack of affordable housing and homelessness [6]. Their children are at greater risk for impaired physical growth, development, and health, poor cognitive functioning and school performance, emotional and behavioural problems, psychiatric disorders, and substance use themselves [7,8]. Despite their best intentions, women with substance abuse issues are at risk for a wide range of parenting deficits [9]. Parenting can be operationalized as skills (e.g., interacting sensitively, facilitating sleeping and eating routines), attitudes (e.g., empathy, positive approaches to behaviour guidance), knowledge (understanding child development), or capacity (e.g., maternal custody, lack of need for child protection services involvement). Parenting among mothers with substance abuse issues may be impaired by the primacy of satisfying their addiction over the welfare of themselves and their children, the emotional lability that is associated with intoxication or withdrawal, the impairment from chronic drug use, and their consequent unavailability to their children [9]. Further, women with substance abuse issues often have high levels of comorbid psychopathology and personality problems [10-13], which can impair emotional responsiveness and cognitive abilities and negatively impact parenting [9].

As maternal substance abuse is a growing problem, there is an urgent need to identify effective interventions. Treatment for mothers with substance abuse issues and their children may represent an important opportunity for breaking the intergenerational cycle of addiction and dysfunction and improving parenting. However, women with substance abuse issues report difficulties using conventional systems of care (for reasons including fear of losing custody of children, guilt, stigma, and lack of transportation), and request comprehensive services provided in a caring, 'one-stop' setting [14]. Given the barriers, risks, and outcome implications, researchers, clinicians, and policy makers recommend that substance abuse treatment programs address women's needs as well as their children's needs through comprehensive, integrated services in centralized settings for both women and children [14]. This recognition has resulted in the development of numerous integrated treatment programs (those that include on-site pregnancy-, parenting-, or child-related services with addiction services), both residential and outpatient. Integrated residential programs or "therapeutic communities" offer long-term (15-18 months) treatment services to women and their children. Both types of programs typically are comprehensive and include group and individual addiction treatment, maternal mental health services, trauma treatment, parenting education and counseling, life skills training, prenatal education, medical and nutrition services, education and employment assistance, child care, children's services, and aftercare.

Parenting is an important outcome of intervention because it impacts child outcomes [15]. Studies of parenting interventions with other at-risk populations have shown that improving parenting can improve outcomes for children [16-18]. For example, early prevention programs designed to enhance protective factors (i.e., positive parent-child interaction and parenting behaviour) and reduce risk factors (e.g., hostile, negative, or overreactive parenting) prevent later disruptive behaviour disorders in children and adolescents at risk [19]. Thus, the risks to children of women with substance abuse issues could be minimized with intervention. If intervention for mothers with substance abuse issues is successful in improving parenting outcomes, it may reduce costs (in terms of foster care placement, emergency room visits, medical and psychiatric admissions, child treatment, crime, etc.) and enhance healthcare and social service delivery.

To date, no systematic reviews of quantitative studies of parenting outcomes of integrated programs have been conducted. Gender specific (i.e., women only) substance use treatment was examined in one systematic review and one meta-analysis. In their systematic review of 38 studies, Ashley, Marsden, and Brady [20] found that programs with prenatal care or child care were associated with improved outcomes (substance use, mental health, birth outcomes, employment, and health). Similarly, in their meta-analysis, Orwin, Francisco, and Bernichon [21] concluded that enhancing women-only addiction treatment programs with prenatal care or therapeutic child care added value above and beyond the effects of standard women-only programs. Neither of these reviews specifically focused on integrated programs or examined parenting outcomes, despite the potential implications for prevention, harm reduction, improving public health, and reducing the burden to society.

We examined the effectiveness of integrated programs on parenting outcomes in a systematic review of relevant studies. The specific research questions guiding this systematic review were: 1) Are integrated programs more effective than addiction treatment-as-usual in improving parenting outcomes?; and 2) Are some integrated program characteristics associated with better parenting outcomes than others?

## Methods

### Information sources and literature search

We used three main strategies to identify outcome studies of intervention programs for women with substance abuse issues and their children: online bibliographic database searches, checking printed sources, and requests to researchers (cf., [22]). First, we searched relevant bibliographic databases (PsycINFO, MedLine, PubMed, Web of Science, EMBASE, Proquest Dissertations, Sociological Abstracts, and CINAHL) for studies published in English from 1990 to May 2011, using a subject heading and keyword search for the terms "substance abuse (or substance use or addict* or alcohol*) and intervention (or treatment or therapeutic or rehab*) and women (or mother) and child (or infant) and mental health and prenatal (or parent*), singly and in combination. Secondly, we examined reference lists of retrieved articles for potentially relevant documents. In addition, we manually searched relevant journals in the area (*Addiction, Addictive Behaviours, International Journal of the Addictions, Journal of Drug Issues, Journal of Psychoactive Drugs, Journal of Substance Abuse, Journal of Substance Abuse Treatment, Journal of Substance Use, and Substance Use and Misuse*). Documents that appeared to be relevant on the basis of titles or abstracts were retrieved. Finally, we searched for grey literature (technical reports, clinical trials registry, unpublished data). All researchers identified through these searches, as well as researchers presenting at relevant conferences identified using Google and Cross Currents (Upcoming Events), were contacted by email to request any relevant published or unpublished data. Of the 200 researchers identified and emailed, 48% responded and 28 additional studies were identified.

### Eligibility criteria and study selection

Eligibility criteria were based on our working definition of integrated programs being substance abuse treatment programs that provide comprehensive services that address substance abuse as well as maternal and child well being through prenatal services, parenting programs, child care, and/or other child-centred services in a centralized setting. Therefore, we included studies in our larger systematic review if all of the following criteria were met:

1) all participants were women who were pregnant or parenting;

2) all participants had substance abuse problems at baseline;

3) the treatment program included at least one specific substance use treatment (e.g., individual or group therapy, methadone) and at least one parenting or child (< 16 years) treatment service (e.g., prenatal care, child care, parenting classes);

4) the study design was randomized, quasi-experimental, or cohort; and

5) there was quantitative data on parenting or other outcomes as part of the larger study (length of stay, treatment completion, maternal substance use, maternal well-being, or child well-being).

### Data extraction

Upon completion of the literature search, we developed a codebook based on theoretical treatment models, literature review, and data availability. We collected data on dependent variable characteristics (type of outcome measure, type of data), and outcome statistics (e.g., F value, p value) and coded study context (author, document date, type of document, country), methodology (sample size, attrition, study design), participant characteristics (age, marital status, education, employment, income, substance abuse history, previous substance abuse treatment, mental and physical health, involvement with the legal system), child characteristics (age, custody, involvement with child protection services, positive toxicology at birth), and treatment program characteristics (population served, planned length of treatment, intensity of treatment, location, services). Project staff and investigators pilot tested the codebook, which we revised based on consensus before formally coding the studies. In a coding policy manual, we recorded variables that were added or deleted and decisions regarding clarification of specific variables.

A trained research assistant (AS) coded each study and met frequently with the principal investigator (KM) during the development of the codebook and early stage of coding. Both AS and KM coded 20% of studies. We calculated Cohen's *Kappa *and percent agreement for all variables. There was 100% agreement for identification of dependent variables and, for client and program variables, 94% mean agreement for continuous variables and a *Kappa *of 0.97 for categorical variables. We resolved discrepancies by consensus.

There were considerable missing data (especially on client characteristics and program services) and limited quantitative data on outcomes (e.g., standard deviations, sample sizes). In an attempt to obtain missing data, we contacted 89 researchers up to three times each. Our attempts to contact researchers occurred throughout the coding process up until data analyses were completed. In total, 79% responded, with 37% providing some additional data (Additional file 1).

### Study quality

To assess the quality of randomized trials, we used the Jadad Scale [23], which is widely used in the medical literature. On the Jadad Scale, studies are rated on a scale from 0 to 5, with the highest possible score (5) given for those with descriptions of the randomization process, an appropriate method of randomization, double-blinding, an appropriate method of double-blinding, and withdrawal and dropouts. To assess the quality of non-randomized studies, we used the Newcastle-Ottawa Scale (NOS; [24]). On the NOS, studies are rated on a scale from 0 to 9 on the basis of three main issues: study group selection, group comparability, and outcome ascertainment. NOS content validity and inter-rater reliability have been established and further evaluation is being conducted [24]. A trained research assistant (AS) and Master's student (JL) coded study quality. Inter-rater reliability, based on 16% (19) of the 121 eligible studies, was high, *Kappa *= 0.81. We resolved discrepancies by consensus.

### Calculating effect sizes

In order to facilitate our summary and comparison of studies, effect sizes were calculated, where possible. We transformed results from each study to the standardized mean difference (Cohen's *d*) and used conventional definitions of effect size (*d*), i.e., small = 0.20 or less (i.e., one fifth or less of one standard deviation difference); medium = 0.50; large = 0.80 (i.e., four fifths or more of one standard deviation difference) [25].

### Program characteristics as moderators

We reviewed studies that examined factors that may have moderated the effect of treatment on outcomes. Specifically, we examined parenting effect sizes of studies in relation to program characteristics (e.g., residential or not, types of program services provided, targeted substance, whether or not children reside), as these potential moderating factors have been examined in previous studies [26]. Also, we reviewed studies comparing two types of integrated programs to examine which specific integrated program characteristics are associated with better parenting outcomes than others.

## Results

### Study selection

**Figure 1:**
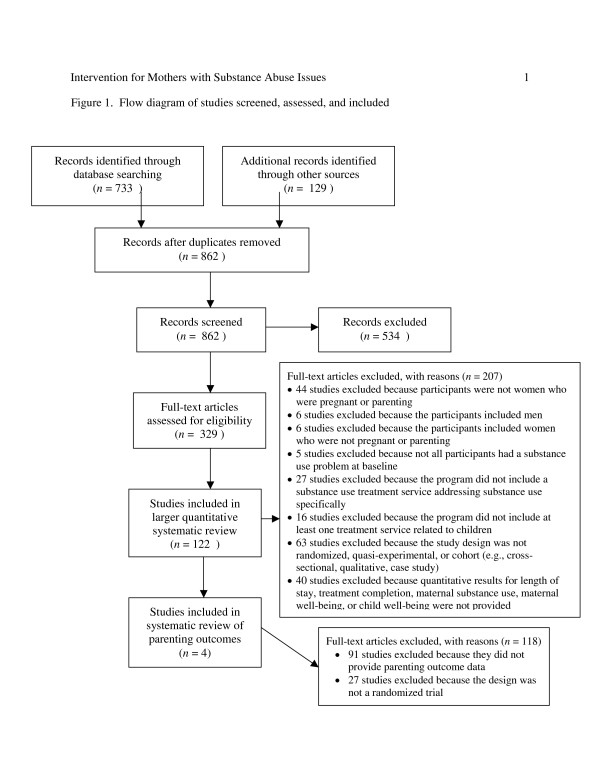
**Flow diagram of studies screened, assessed, and included**.

In total, 329 studies were retrieved and coded for eligibility. Using the eligibility criteria, we excluded 207 and considered 122 studies eligible for inclusion in the larger systematic review. Two randomized trials were not included in our review because their samples included men [27,28] and two other randomized trials were not included because they did not include addiction treatment [29,30]. Based on a random sample of 20% of the studies, inter-rater reliability for eligibility coding was high, *Kappa *= 0.81. We resolved discrepancies by consensus. We estimated the completeness of the search using the capture re-capture method [31]. Based on this method, the estimated number of missing articles is eight (95% confidence interval [CI]: 2, 24), which suggests a 90% capture rate (i.e., the identified studies cover 90% of the search horizon). This reasonably high capture rate suggests that we retrieved a sufficient number of studies to avoid bias in the results of the systematic review. Of the 122 eligible studies, 91 studies did not have quantitative data on parenting outcomes. Of the 31 studies with parenting data, 24 were cohort studies and 3 were quasi-experimental studies [32-34]. Therefore, for the present review, we included four randomized trials [35-39]. See Figure 1 for a flow diagram.

### Study characteristics

Studies varied in terms of assessment times and parenting measures. Parenting outcomes were assessed at varying time points (e.g., at prenatal intake, intake, 12 months postpartum, discharge, 6 weeks after discharge, 6 months after discharge). Three studies included measures of parenting skills (Parent-child Relationship Inventory, Parental Acceptance Rejection Questionnaire, Working Model of the Child Interview, Parent Development Interview, Nursing Child Assessment Satellite Training) and one study examined parenting capacity (child protection services involvement).

One study [35] involved pregnant women and the other three studies involved mothers with children (an average of 2 or 3). The average age was 29-36 years. Most women had experienced trauma, had mental health problems, and were unemployed, single mothers. Client race varied among studies, reflecting the geographic setting. Authors provided little specific information on the children, who were of a wide age range (infants to adolescents). Programs were 3-12 months and had a high dropout rate.

### Study quality

Jadad Scale scores for two of the randomized trials were 3, which is a moderate score [36,37]. Both studies were described as randomized but were not double blind as participants were aware of the treatment allocation. They both provided descriptions of an appropriate method of randomization and withdrawal and dropouts. The Jadad Scale score for one other randomized trial was 2, which is a low score [38,39]. The study was described as randomized and provided a description of withdrawal and dropouts but was not double blind and did not provide a description of an appropriate method of randomization. The Jadad Scale score for another randomized trial was 1, which is a low score [35]. The study was described as randomized but was not double blind and did not provide descriptions of an appropriate method of randomization and withdrawal and dropouts.

### Are integrated programs more effective than addiction treatment-as-usual in improving parenting outcomes?

There were three randomized trials comparing parenting outcomes for clients participating in integrated programs and addiction treatment-as-usual [35-37]. As can be seen in Table 1, for the two studies with data on measures of parenting skills, *d*s ranged from 0.00 to 0.94 and most indicated greater pre-post improvements in scores for integrated programs than addiction treatment-as-usual, but this advantage was typically small. In the one study of child protection services involvement [35], there were no group differences in pre-post changes. Below we provide a narrative review of each of the three studies.

**Table 1:** Randomized trials examining parenting outcomes of integrated programs

Study	n	Groups	Measure	Time point	Effect Size (SE)	p	Treatment Group %	Control Group %	Study Quality
Huber [35]	Treatment:82 Control:77	Integrated residential treatment vs standard outpatient treatment	CPS Involvement	Prenatal (Intake)			22	14	1/5

				1 Year Postpartum			46	47	

Huber [35]	Treatment: 81 Control: 77	Integrated outpatient treatment vs standard outpatient treatment	CPS Involvement	Prenatal (Intake)			17	14	1/5

				1 Year Postpartum			46	47	

Luthar & Suchman [36]	Treatment: 32 Control: 20 Treatment: 28 Control: 19	Standard methadone treatment plus maternal psychotherapy vs. standard methadone treatment	PARQ^b^	Discharge	0.54 (0.29)	0.063			3/5
			
				6-month follow-up	0.57 (0.30)	0.060			
			
			PCRI^c ^- Affective Interaction	Discharge	0.94 (0.30)	0.002			
			
				6-month follow-up	0.54 (0.30)	0.074			
			
			PCRI - Limit Setting	Discharge	0.08 (0.29)	0.779			
			
				6-month follow-up	0.20 (0.30)	0.502			
			
			PCRI - Autonomy	Discharge	0.13 (0.29)	0.649			
			
				6-month follow-up	0.33 (0.31)	0.270			
			
			PCRI - Parenting Support	Discharge	0.00 (0.29)	1.000			
			
				6-month follow-up	0.21 (0.30)	0.481			
			
			PCRI - Parenting Satisfaction	Discharge	0.49 (0.29)	0.090			
			
				6-month follow-up	0.35 (0.30)	0.242			

Luthar et al. [37]	Treatment: 60 Control: 67	Standard methadone treatment plus maternal psychotherapy vs. Standard methadone treatment plus recovery training	PARQ	Discharge	0.23 (0.18)	0.206			3/5
				6-month follow-up	0.13 (0.18)	0.471			
			
			PCRI - Affective Interaction	Discharge	0.11 (0.18)	0.527			
				6-month follow-up	0.15 (0.18)	0.400			

			PCRI - Parenting Satisfaction	Discharge	0.10 (0.18)	0.590			
				6-month follow up	0.18 (0.18)	0.313			

Suchman et al. [38,39]	Treatment: 23 Control: 24	Outpatient substance abuse treatment plus attachment-based parenting intervention vs outpatient substance abuse treatment plus parent education	WMCI^d ^acceptance	Discharge	-0.04 (0.29)	0.886			2/5
			
			WMCI involvement	Discharge	0.13 (0.29)	0.653			
		
			WMCI coherence	Discharge	0.02 (0.29)	0.949			
		
			WMCI openness	Discharge	0.04 (0.29)	0.897			
		
			WMCI sensitivity	Discharge	0.50 (0.30)	0.092			
		
			WMCI quality of representations	6-week follow-up	0.22 (0.29)	0.455			
		
			NCAST^e ^caregiving behavior	Discharge	0.70 (0.30)	0.020			

				6-week follow-up	0.12 (0.29)	0.678			

			PDI^f ^reflective functioning	Discharge	0.61 (0.30)	0.042			

				6-week follow-up	0.22 (0.29)	0.452			

			PDI self-focused reflective functioning	6-week follow-up	-0.22 (0.29)	0.452			

			PDI child-focused reflective functioning	6-week follow-up	0.03 (0.29)	0.93			

^a^CPS = Child Protection Services

^b^PARQ = Parental Acceptance Rejection Questionnaire - Maternal report of maltreatment risk

^c^PCRI = Parent-child Relationship Inventory

^d^WMCI = Working Model of the Child Interview

^e^NCAST = The Nursing Child Assessment Satellite Training

^f^PDI = Parent Development Interview

As part of the Washington State MOMS project, Huber [35] randomly assigned pregnant women with substance abuse issues to an integrated residential program, an integrated outpatient program, or a standard outpatient program. The integrated programs included prenatal care, maternal health care, parenting education and support, and children's services. Huber [35] found no group differences in the percentage of clients involved with child protection services. Child protection services involvement appeared to increase for all groups from intake in the prenatal period to one year postpartum, but did not report *p *values.

Luthar and Suchman [36] randomly assigned mothers (of children under 16 years old) at three methadone clinics in New Haven, Connecticut, to standard treatment or standard treatment plus a relational psychotherapy mothers' group. Standard treatment included addiction counseling, pharmacological intervention (methodone), case management to assist with basic needs such as housing, welfare benefits, and legal aid. Maltreatment risk was assessed by maternal report on the Parental Acceptance Rejections Questionnaire and parenting skills were assessed using the Parent-child Relationship Inventory. At the end of the 6-month treatment, mothers in integrated treatment had significantly more improved affective interaction scores than mothers in standard treatment and there was a trend toward more decreased maltreatment risk scores and more improvement in parenting satisfaction scores for mothers in integrated treatment than for mothers in standard treatment. At 6-month follow-up, group differences were not significant but there was a trend toward more decreased maltreatment risk scores and more improvement in affective interaction scores for mothers in integrated treatment than for mothers in standard treatment. Limitations of this study include the small sample size, unknown variability in the standard treatment, changes to the integrated program over the course of the study, and dosage differences between the two groups.

With another sample, Luthar et al. [37] randomly assigned mothers (of children under 16 years old) at three methadone clinics in New Haven, Connecticut, to standard treatment plus recovery training or standard treatment plus a relational psychotherapy mothers' group. Maltreatment risk was assessed by maternal report on the Parental Acceptance Rejections Questionnaire and parenting skills were assessed using the Parent-child Relationship Inventory. At the end of the 6-month treatment and at 6-month follow-up, mothers in integrated treatment had more decreased maltreatment risk scores and more improvements in affective interaction and parenting satisfaction, but group differences were not significant.

### Are some integrated program characteristics associated with better parenting outcomes than others?

Examination of parenting effect sizes (where available) among the 31 studies with parenting outcome data suggested that residential programs appeared to have larger effects than outpatient programs and programs with a maternal mental health service appeared to have larger effects than programs that did not offer a maternal mental health service. Only two cohort studies and one randomized trial specifically examined factors associated with parenting outcomes. Kern et al. [40] examined correlations between changes in various domains of parenting stress over the course of treatment and reduction in depressive symptoms. Findings indicated that reduction in depressive symptoms was significantly correlated with improvements in parenting competence, isolation, attachment, and role restriction. Knight and Wallace [41] found that when children resided in the treatment facility, mothers were five times more likely to have custody of their children at the end of treatment.

In a study comparing two integrated programs, Suchman and colleagues [38,39] randomly assigned mothers (of children under 3 years old) in outpatient substance abuse treatment to the Mothers and Toddlers Program (MTP; an attachment-based parenting intervention) or the Parent Education Program (PE; case management and child guidance pamphlets). Quality of mental representations of parenting was assessed using the Working Model of the Child Interview, caregiving behavior was assessed using the Nursing Child Assessment Satellite Training, and maternal reflective functioning was assessed using the Parent Development Interview. As can be seen in Table 1, *d*s ranged from -0.22 to 0.70 and most indicated greater improvements in scores for attachment-based parenting intervention than parent education, but this advantage was typically small. At the end of the 3-month treatment, mothers in the MPT had significantly more improved scores for caregiving behavior and reflective functioning and a trend for more improved sensitivity score than mothers in the PE group. At 6-week follow-up, there were no significant group differences in improvements in scores.

## Discussion

The purpose of this systematic review was to examine the effectiveness of integrated treatment programs on parenting outcomes. In three randomized trials, most effects favored integrated programs over addiction treatment-as-usual and most effects were small. As such, available evidence suggests that integrated programs may be associated with a small advantage over addiction treatment-as-usual in parenting skills outcomes. There were no group differences reported for changes in the proportion of clients involved with child protection services. Unfortunately, there were no randomized trials comparing integrated programs to addiction treatment-as-usual on parenting attitudes, parenting knowledge, or maternal custody. In the three studies that examined factors associated with treatment effects, parenting improvements were associated with attachment-based parenting intervention, children residing in the treatment facility, and improvements in maternal mental health [38-41].

### Implications

The findings of this systematic review are consistent with those reported in previous reviews of substance abuse treatment for women [20,21], meta-analyses of integrated programs showing their positive impact on maternal mental health and birth outcomes [42,43], qualitative studies in which women stated that integrated programs helped them gain insight into intergenerational influences on parenting, how to strengthen emotional bonds with children, and use positive discipline techniques [44], studies of parent interventions with parents (mothers and fathers) in methadone maintenance treatment [27,28], mothers in drug court [29], and other at-risk populations [16]. Results from this systematic review are important given the risks for poor outcomes in children of women with substance abuse issues [45]. The findings suggest that the risks to parenting could be minimized with intervention, which could have long-term impact. For example, integrated programs may improve parenting, which has been shown to reduce the risk of child maltreatment [18]. Even though the advantage of integrated programs over addiction treatment-as-usual may be small, it could have a potentially large impact on the associated financial and human burden in this vulnerable population (e.g., it may reduce the need for foster care placement, child treatment, psychiatric admissions, crime, etc.).

### Limitations

There were a number of challenges encountered in completing this systematic review that highlight current limitations in research on integrated treatment programs. First, among the 122 studies examining outcomes of integrated programs there were only 31 with data on parenting outcomes, despite the fact that improving parenting is often a stated goal of integrated programs. Among the studies reporting parenting outcome data, few were comparison group studies. While not included in the present review, 24 cohort studies assessing parenting outcomes were identified in the literature search. This type of study design provides information about parenting outcomes for women in integrated treatment but does not provide a comparison group that enables one to determine if these outcomes are significantly better than those for women who participated in other types of treatment. Despite the limited number of studies included in the systematic review, we are confident that the search was not biased. We used several approaches to mitigate potential bias, including our attempts to identify grey literature by searching databases that include unpublished studies and contacting researchers for unpublished data as well as our use of the capture re-capture method to estimate the completeness of the literature search (identified studies covered 90% of the search horizon, suggesting that a sufficient number of studies were retrieved to avoid bias).

A second limitation of the present systematic review is that study quality was not high, as is typical of the substance abuse treatment field generally [26]. Studies included in the systematic review were of low to moderate quality, although it was unclear if the scores reflected study design per se or the reporting of study quality elements.

A third limitation is that studies had small samples and relatively few parenting outcome measures. The randomized trials comparing integrated programs to addiction treatment-as-usual did not involve observational measures of parenting, which may be more objective and valid than self-report measures. Also, these studies did not involve measures of some important areas of maternal functioning that can be impacted by substance abuse, such as maternal responsiveness, sensitivity, and reflective functioning, nor did they involve longitudinal follow-up on parenting or an assessment of cost effectiveness.

Fourthly, missing data limited our review. Information on research methods and data needed to calculate effect sizes precluded meta-analysis and hampered attempts at assessing study quality. Often program, client, and study characteristics that might moderate treatment outcomes was not available. Moderator analyses can have important practice implications, however, specific recommendations (e.g., regarding specific intervention strategies or specific subpopulations to target such as mothers of children in or out of foster care or mothers of younger or older children) await further research with better reporting to allow meta-analysis of variables that impact outcomes (c.f., [26]).

### Recommendations for future research

More high quality studies comparing integrated programs to addiction treatment-as-usual are needed, especially studies of programs that target parent-child interaction with mothers of young children examining a variety of parenting outcomes. A multi-site study could address statistical limitations inherent to small samples. The most rigorous design would be a randomized trial, but this may be challenging in the world of real-life service provision. Further, the examination of moderators is critical, given the variability in clients served and services offered. Also, examination of moderators would help identify effective components of intervention and ultimately examine what works best for whom under what circumstances. As some effects may not be immediately evident, follow-up for at least two years (or, ideally, longer) would be advisable. Linear regression, generalized estimating equations, or linear growth curve modelling could be used to analyze parenting outcomes with group and other variables as predictors (e.g., maternal and child characteristics, program components), as well as the impact of mediators and moderators over time [46]. The propensity score method could be used to address the potential problem of baseline differences between groups [47]. Evaluation of parenting outcomes could be improved through the use of observational measures, with videotaped observations coded by research assistants blind to group status. Ensuring the availability of essential information to assess study quality and describe studies in future reviews could be accomplished by improvements in the editorial review process and creation of a registry of funded studies that would require submission of standard information (such as the Cochrane Collaboration on health care intervention), as has been recommended previously (e.g., [26]).

## Conclusions

The findings from this systematic review suggest that integrated programs for women with substance use issues and their children may be associated with positive impacts on parenting skills and capacity. These findings are encouraging in terms of the preventive potential for breaking the cycle of addiction, dysfunctional parenting, and poor outcomes for many vulnerable children. Consistent with the recommendations for research synthesis of Cooper, Hedges, and Valentine [48], this review addresses an important and under-recognized, yet growing, area of research. The findings suggest the potential promise of integrated programs and highlight research gaps in study design, quality, and reporting practices. Future research involving prospective longitudinal studies with comparison group designs, larger samples, and full descriptions of the target population and the intervention program is recommended. To our knowledge, this systematic review is the first to examine the impact of integrated programs on parenting outcomes. Given that approximately one third of substance abusers are women of child-bearing age [49], substance use among pregnant and parenting women is a serious problem for the child welfare system and a major public health concern, and the burden of suffering due to maternal substance abuse is great, the findings from this review are noteworthy and support the need for more high quality research on integrated treatment programs for women with substance abuse issues and their children. The effectiveness of integrated programs warrant further exploration and investigation, as the implications of their wide-spread implementation may include reduced costs to taxpayers, increased access, and more positive outcomes for mothers and children.

## Abbreviations

NOS: Newcastle-Ottawa Scale;

## Competing interests

The authors declare that they have no competing interests.

## Authors' contributions

AN conceived of the study, participated in the design of the study, interpreted the data and led preparation of the manuscript. KM conceived of the study, participated in the design of the study, supervised the literature search, led the development of the codebook, supervised the coding, performed reliability coding, and drafted sections of the manuscript. WS participated in the design of the study and the development of the codebook and contributed to sections of the manuscript. LT participated in the design of the study, the development of the codebook, and data analysis, and contributed to sections of the manuscript. JH participated in the development of the codebook and interpretation of the data. AS conducted the literature search and coding and participated in the development of the codebook and interpretation of the data. All authors commented on drafts of the manuscript and approved the final edition.
